# Serum copper decrease and cerebellar atrophy in patients with nitrous oxide-induced subacute combined degeneration: two cases report

**DOI:** 10.1186/s12883-021-02496-y

**Published:** 2021-12-04

**Authors:** Jie Cao, Lusen Ran, Chenchen Liu, Zhijun Li

**Affiliations:** Department of Neurology, Tongji Hospital, Tongji Medical College, Huazhong University of Science and Technology, Wuhan, 430030 China

**Keywords:** Nitrous oxide, Subacute combined degeneration, Serum copper, Cerebellar atrophy, Cognitive decline

## Abstract

**Background:**

Subacute combined degeneration (SCD) is a neurological complication commonly associated with vitamin B_12_ deficiency. It can result from nitrous oxide (N_2_O) abuse and cause neuropsychiatric symptoms. However, there has been no literature regarding alterations of serum copper and cerebellum in SCD patients.

**Case presentation:**

We reported two cases of young SCD patients with histories of N_2_O abuse. In these cases, elevated homocysteine, macrocytic anemia, spinal cord abnormalities, and peripheral nerve injuries were detected. In addition, decreased serum copper level and cerebellar atrophy were reported for the first time. The patients’ symptoms improved after withdrawal of N_2_O exposure and vitamin B_12_ supplements.

**Conclusion:**

We reported two SCD cases with serum copper alteration and cerebellar atrophy after N_2_O abuse for the first time. These might be crucial complements to the diagnosis of SCD.

## Background

Subacute combined degeneration (SCD) is a neurological disease usually induced by vitamin B_12_ deficiency [1–4]. Previous studies demonstrate that SCD can result from recreational nitrous oxide (N_2_O) abuse and cause various neuropsychiatric disorders [5–9]. However, no literature has been concerned about the serum copper and cerebellar changes through the diagnostic process. Here for the first time, we report two cases of SCD patients induced by N_2_O abuse with decreased cupric ion and cerebellar atrophy, intending to explore new clinical features of the disease.

## Case presentation

### Case 1

A 20-year-old woman complained of weakness and numbness in both lower limbs for 10 days. She had inhaled N_2_O gas for over a year before admission. Physical examination showed decreased muscle strength in both lower limbs (grade 4/5) and deep sensational disturbances in extremities. The muscle tension was normal, and the tendon reflex of the limbs was symmetrically active. The patient also had a “drunken” gait, “explosive speech”, nystagmus, and cerebellar ataxia (positive Romberg sign, abnormal finger-to-nose and heel-to-shin tests with eyes open or closed).

**Fig. 1:**
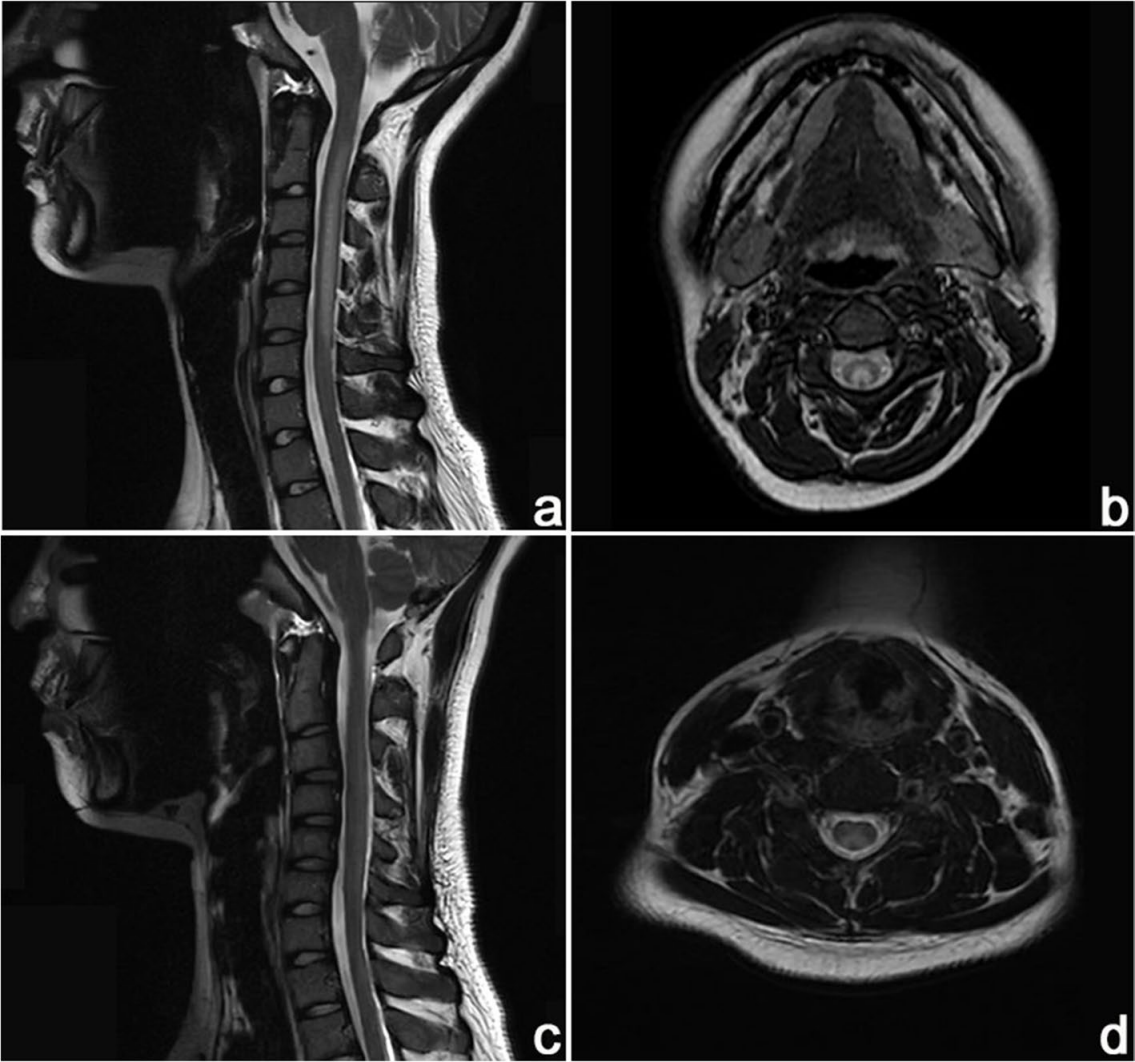
Spinal MRI findings of the two patients. In case 1, **(a)** sagittal T_2_-weighted imaging showed symmetrical hyperintensities from C2 to C5 segment in both posterior and lateral columns of the spinal cords, and **(b)** axial T2-weighted imaging revealed typical inverted “V”- shaped lesion. In case 2, **(c)** sagittal T_2_-weighted imaging showed symmetrical hyperintensities from C2 to T1 segment in both posterior and lateral columns of the spinal cords, and **(d)** axial T_2_-weighted imaging revealed typical inverted “V”- shaped lesion

**Fig. 2:**
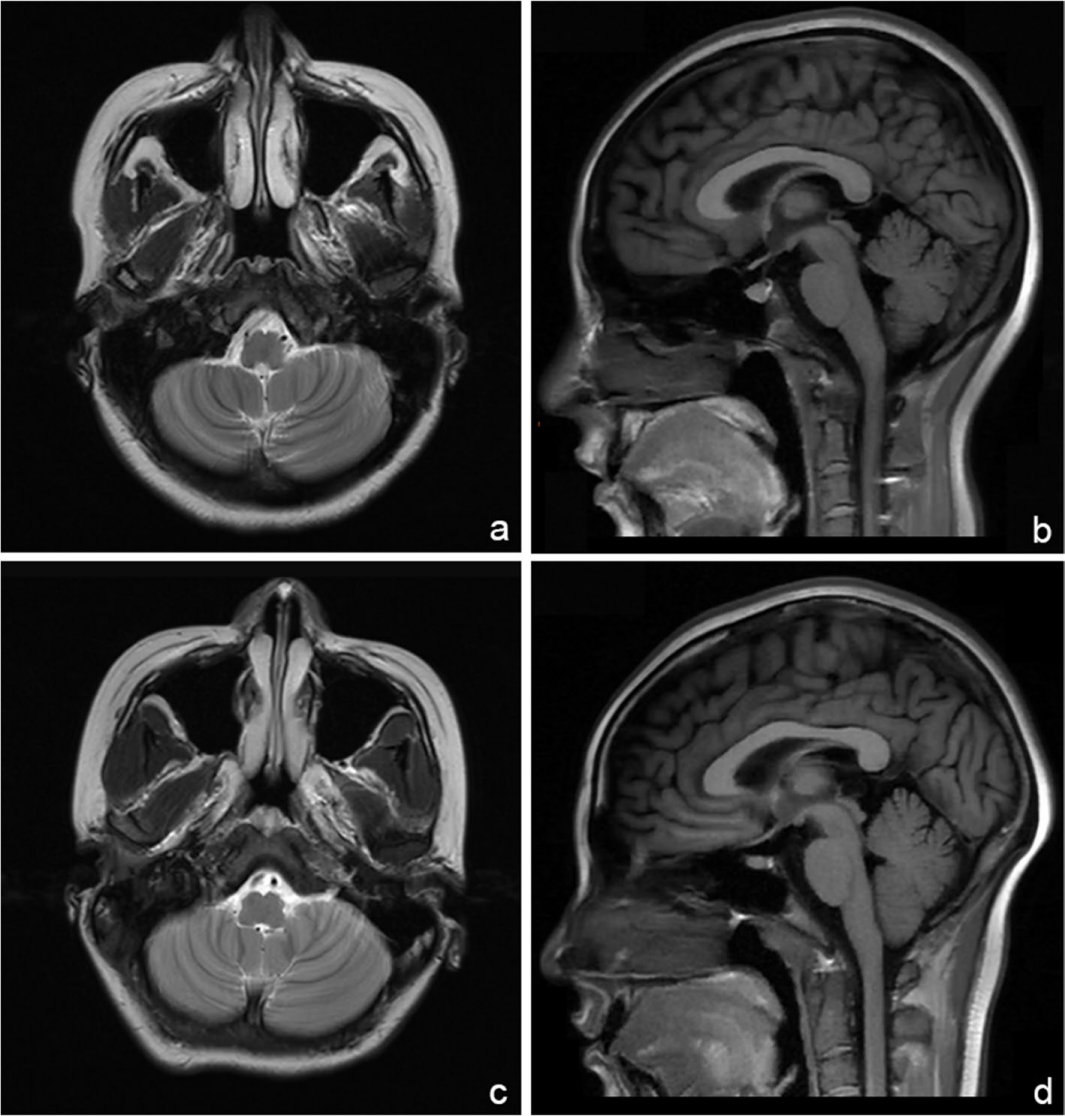
Brain MRI findings of the two patients. Axial T2-weighted imaging and sagittal T1-weighted imaging showed enlarged cerebellar sulci in **(a, b)** case 1 and **(c, d)** case 2 relatively, indicating cerebellar atrophies

Laboratory examinations showed increased serum vitamin B_12_ (>1525 pg/mL, normal range 180–914 pg/mL) and decreased folic acid (3.54 ng/mL, normal range > 4.00 ng/mL) levels. The serum homocysteine (15.5 umol/L, normal range 6.0–14.0 umol/L) level was increased, and the methylene tetrahydrofolate reductase (MTHFR) genotype reveals normal metabolic capacity. The blood routine test presented macrocytic anemia. The detection of metal ions revealed declined serum copper level (10.5 umol/L, normal range 12.6–24.4 umol/L), and serum zinc level was within the normal range. The routine, biochemical, immunological, and etiological examination in cerebral spinal fluid appeared normal. Other laboratory examinations were within the normal range. The motor and sensitive nerve conduction velocities and electromyography showed decreased motor and sensitive nerve conduction velocities in extremities and sensorimotor polyneuropathy. Spinal MRI revealed abnormal signals in posterior and lateral columns of the spinal cords, presented as inverted “V”- shaped lesions (Fig. 1a, b). Brain MRI displayed slight cerebellar atrophy (Fig. 2a, b). Besides, she had poor cognitive behavior with a mini-mental state examination (MMSE) score of 18.

The patient was diagnosed with SCD and asked to stop N_2_O inhalation. Ten days after cobalamin, folate, and symptomatic treatment, the homocysteine level returned to normal. Four days later, the patient was discharged with reduced limb numbness and slightly increased muscle strength of both lower limbs than before.

The patient was asked to continue taking cobalamin and folate after discharge. At 2-week’s follow-up, her walking function and cognitive behavior improved (MMSE score = 22). Three months after discharge, the patient got recovered in the muscle strength and gait, but still felt numbness in toes. The patient completely recovered in the follow-up at 6 and 12 months after discharge respectively. The levels of serum vitamin B_12_, folate, copper, and hemoglobin were all in the normal range at 3, 6, and 12 months after discharge.

### Case 2

A 19-year-old woman complained of speech disorders, weakness, and numbness of her lower limbs for 2 weeks. She had inhaled N_2_O gas for nearly one year and the dose was largely increased in the recent two months. Half a year after inhalation, she began to take vitamin B complex irregularly. The patient hummed and talked discontinuously, and also presented intermittent hallucinations during the conversation on admission. Physical examination showed decreased muscle strength of lower limbs (grade 4/5), declined muscle tension, hypoesthesia, and deep sensational disturbances in extremities. The patient also had a “drunken” gait, “explosive speech”, nystagmus, and cerebellar ataxia (positive Romberg sign, abnormal finger-to-nose and heel-to-shin tests with eyes open or closed).

Laboratory examinations showed increased serum vitamin B_12_ (>1515 pg/mL, normal range 180–914 pg/mL) level and macrocytic anemia. The serum homocysteine (22.6 umol/L, normal range 6.0–14.0 umol/L) level was increased, and the methylene tetrahydrofolate reductase (MTHFR) genotype revealed normal metabolic capacity. We were also concerned about the copper metabolism and found decreased serum copper (10.1 umol/L, reference range 12.6–24.4 umol/L) and ceruloplasmin (0.194 g/L, reference range 0.22–0.58 g/L) levels. The serum zinc level was within the normal range. The routine, biochemical, immunological, and etiological examination in cerebral spinal fluid appeared normal. Other laboratory examinations were within the normal range. The motor and sensitive nerve conduction velocities and electromyography showed decreased motor and sensitive nerve conduction velocities in extremities and sensorimotor polyneuropathy. Spinal MRI revealed abnormal signals in posterior and lateral columns of the spinal cords, presented as inverted “V”- shaped lesions (Fig. 1c, d). Brain MRI indicated slight cerebellar atrophy (Fig. 2c, d). She had poor cognitive behavior with an MMSE score of 14 on admission.

The patient was diagnosed with SCD and asked to stop N_2_O inhalation. After cobalamin, folate, and symptomatic treatment for 9 days, the homocysteine level returned to normal. She was discharged 2 days later with better cognitive performances (MMSE score increased to 28) and sensational function.

The patient was asked to continue taking cobalamin and folate after discharge. At 2-week’s follow-up, she had much fewer hallucinations with the MMSE score of 30, but still walked unsteadily. Three months after discharge, the patient got recovered in the muscle strength and superficial sensation, but still had slight ataxia. The patient completely recovered in the follow-up at 6 and 12 months after discharge respectively. The levels of serum vitamin B_12_, folate, copper, and hemoglobin were all in the normal range at 3, 6, and 12 months after discharge.

## Discussion and conclusions

In recent years, N_2_O has gained increasing popularity worldwide especially among young people due to its euphoric effects [10, 11]. According to previous studies, it is not unusual that N_2_O can cause cobalamin deficiency and SCD [5, 7, 11–13]. Although serum vitamin B_12_ is a most convenient and accessible laboratory approach to evaluate cobalamin deficiency, it is to note that quite a few SCD patients are detected with normal or elevated serum vitamin B_12_ [14–17]. In fact, a growing number of N_2_O addicts are beginning to take vitamin B_12_ irregularly before admission. Even if patients do not take the supplements after inhaling N_2_O, serum vitamin B_12_ levels may also be in the normal range [18]. A meta-analysis manifests that about a third of SCD patients are detected with no-low serum vitamin B_12_ levels [19]. In a systematic review, Garakani et al. indicate that only less than half of adolescents with N_2_O abuse disclosed low levels of serum vitamin B_12_ [18].

These bring about challenges to traditional diagnostic thought that the total serum vitamin B_12_ is the most preferential indicator to diagnose SCD. Literature suggests that a SCD patient with a normal or elevated level of serum vitamin B_12_ may still have a vitamin B_12_ shortage in the body, which is also described as functional vitamin B_12_ deficiency [20]. It has been gradually realized that serum vitamin B_12_ does not reflect genuine “active” vitamin B_12_ level. Compared with serum vitamin B_12_, there are more sensitive markers to reveal cobalamin deficiency at the tissue level, such as methylmalonic acid and homocysteine [21, 22]. Therefore, it is reasonable to use combined serological indicators for assessing vitamin B_12_ deficiency. As is shown in our cases, the declined level of serum vitamin B_12_ is not a compulsory element for SCD diagnosis, while an elevated level of homocysteine is helpful for the evaluation of functional vitamin B_12_ deficiency.

Copper is one of the trace elements which plays an important role as a component of enzymes in the nervous system [23]. As an essential element of methionine synthase in the methylation cycle, the lack of serum copper leads to dysfunction of methionine synthase, which can cause demyelination that resembles SCD [24, 25]. The pathological copper deficiency was usually caused by insufficient storage (preterm and infants), insufficient intakes or malabsorption (diet causes, chronic diarrhea, celiac disease, Crohn’s disease, long-time parenteral nutrition, intestinal surgery, and excessive zinc), increased demands (pregnancy, lactation and wound healing), increased losses (major burns and renal replacement therapy), or hereditary diseases (such as Menkes disease) [26, 27]. As our cases had clear histories of N_2_O abuse and none of the above causes were involved, we suspected a possible link between the copper deficiency and N_2_O abuse. So far as we know, this is the first report concerning the alteration of serum copper in SCD following N_2_O abuse. To our knowledge, there has been no statement why N_2_O abuse could cause copper deficiency in previous articles. We speculated the possible reasons are as follows: Copper is a cofactor for a variety of biological reactions and thought to be necessary for methionine synthase and S-adenosylhomocysteine hydrolase, which play vital roles within the methylation cycle [10]. N_2_O can oxidize the cobalt ion which attenuates the function of methylcobalamin, inhibits methionine synthase, and elevates the concentration of homocysteine [5]. This might be followed by a decrease in the activity of S-adenosylhomocysteine hydrolase and a consequent decline in copper concentration by feedback regulations. However, more studies are needed to uncover the possible mechanism.

SCD generally affects the spinal cord and peripheral nerves, but rarely impacts the brain [28, 29]. The essential brain pathological manifestation involves demyelination in the cerebral white matter [30]. However, there has been no report of cerebellar atrophy following SCD up to the present. To exclude cerebral atrophy induced by other diseases, the histories of our patients were asked repeatedly. Any symptoms in the nervous system, like speech disorder, weakness, clumsiness, abnormal sensation, unsteady gait, dizziness, etc., did not exist before N_2_O abuse. Through asking detailed histories, present causes for cerebral atrophy, like multiple system atrophy, intracranial infection, paraneoplastic syndrome, hereditary diseases (such as spinocerebellar degeneration, Friedreich ataxia, and dentatorubral-pallidoluysian atrophy), alcohol/drug abuse, traumatic brain injury, cerebrovascular disorders, etc. [31–33], were also excluded. As both of the young patients were with no medical or family histories before N_2_O inhalation, it was unlikely that the atrophies of our cases were caused by other reasons. We thus suspect that these changes resulted from N_2_O abuse. Our article is the first report involving cerebellar atrophy following N_2_O abuse, which diversifies brain changes in SCD patients. Previous literature argues that the cerebellum has a smaller requirement for vitamin B_12_, which may indicate smaller storage of vitamin B_12_ in the cerebellum. Therefore, the cerebellum may be more sensitive to chronic vitamin B_12_ deficiency. This is perhaps a potential cause of cerebellum ataxia under a long-course vitamin B_12_ deficiency [34, 35]. Although the mechanism is vague, it highlights the harmfulness of N_2_O abuse as a long-term recreational purpose from neuroimaging studies.

Our article provided potential importance to sophisticate the diagnosis of SCD. Here we reported two cases of young SCD patients after N_2_O abuse, who were both detected with elevated vitamin B_12_, declined serum copper, and cerebellar atrophy. It is supposed that the serum copper and cerebellar alteration could be auxiliary diagnostic indicators of SCD. These may be meaningful for comprehensive diagnoses of SCD patients.

## Data Availability

The data that support the findings of the current study are available form the corresponding author upon reasonable request.

## Abbreviations

SCD: Subacute combined degeneration
N_2_O: nitrous oxide
MTHFR: methylene tetrahydrofolate reductase
MMSE: Mini-mental State Examination
